# Correlation Between Coronavirus Disease 2019 and Olfactory Dysfunction

**DOI:** 10.3389/fpubh.2022.843850

**Published:** 2022-03-22

**Authors:** Yufeng Mao, Bin Ye, Cui Fan, Jichang Wu, Beilei Wang, Yilin Shen, Zhihong Shi, Mingliang Xiang

**Affiliations:** Department of Otolaryngology and Head and Neck Surgery, Ruijin Hospital, Shanghai Jiao Tong University School of Medicine, Shanghai, China

**Keywords:** COVID-19, etiology, olfactory dysfunction, pathogenic mechanism, viral upper respiratory tract infection

## Abstract

A great number of patients with Coronavirus Disease 2019 (COVID-19) experience olfactory dysfunction, typically of a short duration and with a high incidence rate, during the early stages of infection with severe acute respiratory syndrome coronavirus 2 (SARS-CoV-2). This kind of olfactory dysfunction appears more likely in young people and women. This study presents a review of the clinical features and pathogenic mechanism of the olfactory dysfunction related to SARS-CoV-2 infection, aiming to provide a clinical reference for the diagnosis, differential diagnosis, treatment, and prevention of olfactory dysfunction in COVID-19 patients.

## Introduction

Research shows that binding of the spike proteins located on the surface of severe acute respiratory syndrome coronavirus 2 (SARS-CoV-2) to functional angiotensin-converting enzyme 2 (ACE2) receptors on the surfaces of host cells facilitates virus entry, replication, assembly, and the subsequent infection of other cells (1). The distribution of ACE2 receptors is organ-specific. Besides their high expression in lung tissues, they are also expressed in many extrapulmonary organs, such as nasal mucosa. Waradon et al. found that ACE2 receptors are expressed at a high level in nasal epithelial cells, peculiarly in goblet and ciliated cells, *via* single-cell transcriptome analysis (2). Therefore, although patients with coronavirus disease 2019 (COVID-19) normally experience symptoms such as fever, cough, and shortness of breath as the first symptoms of infection, studies have also indicated that a portion of COVID-19 patients show symptoms of the ear, nose, and throat (ENT), such as olfactory dysfunction, nasal obstruction, runny nose, sore throat, and sore ears, and that these are sometimes the only visible symptoms in patients infected with SARS-CoV-2.

At the start of the COVID-19 pandemic, the public, medical personnel, and researchers generally focused on the known signs and symptoms of SARS-CoV-2 infection, such as fever of an unknown cause, lung infection, and ground-glass opacities on X-ray imaging, while ENT symptoms were ignored. However, with the accumulation of clinical cases and continued investigation, a clear correlation between COVID-19 and ENT symptoms has gradually emerged. It has also been reported successively that COVID-19 may cause nervous functional disorders, including hyposmia and anosmia, in patients.

At present, control of the COVID-19 pandemic remains inadequate. Focusing on COVID-19, this article reviews studies on the correlation between COVID-19 and olfactory dysfunction, compares and analyzes the similarities and differences between olfactory dysfunction caused by SARS-CoV-2 infection and olfactory dysfunction caused by other viral infections, and discusses possible mechanisms to provide a theoretical reference for the early screening and diagnosis of COVID-19, as well as the identification and treatment of olfactory dysfunction.

## Olfactory Dysfunction

When the odorant molecule is combined with the olfactory receptors of the cilium epithelium of the olfactory nerve, the olfactory nerve is activated and releases transmitters to generate nerve impulses. In this way, the nerve signal is transmitted along the olfactory nerve to the olfactory bulb, and then through the olfactory bulb to the higher olfactory center to stimulate the olfactory cortex, finally producing olfaction. As such, olfactory dysfunction refers to partial or total hyposmia or anosmia.

There are a number of factors that cause olfactory dysfunction, however, the most common is an upper respiratory tract infection (URTI) of viral origin. More than 200 kinds of viruses are known to be able to cause URTIs, with rhinoviruses (RVs), coronaviruses (CoVs), influenza viruses (IVs), and para-influenza viruses (PIVs) being the common culprits. Suzuki et al. clinically analyzed nasal discharge of patients with olfactory dysfunction secondary to URTIs and identified the viruses in them, confirming that RVs, CoVs, PIVs, and the Epstein–Barr virus can be causes of olfactory dysfunction (3).

Although, compared to reports of other common symptoms of URTIs, reports of olfactory dysfunction are limited in the COVID-19, and more and more studies have focused on the olfactory dysfunction caused by SARS-CoV-2 infection. These reports indicate that there may be differences in the incidence, onset time, duration, and mechanism of the olfactory dysfunction caused by SARS-CoV-2 and that caused by other common URTI pathogens.

### Regularity of the Olfactory Dysfunction Caused by COVID-19

Since SARS-CoV-2 emerged in China at the end of 2019, there have been a number of reports that a portion of COVID-19 patients experience olfactory dysfunction. Elibol conducted a retrospective study to collect patients' medical history and symptoms, among which symptoms of the head and neck, including anosmia and/or ageusia, sore throat, cough, nasal congestion, earache, tinnitus, hearing loss, oral problems, dizziness, hoarseness, and other symptoms, were chief. Of the 155 patients with confirmed COVID-19, 89 (57.4%) were found to have otolaryngological symptoms, including 55 (35.4%) with anosmia (4). Tong et al. screened the literature, excluding case reports and reviews, and finally found 10 studies on olfactory dysfunction published in 2020 from a total of 9 countries, including China, the United States, the United Kingdom, Spain, and France; two studies were multinational. Peter used a random-effects model to perform a meta-analysis, where a total of 845 patients (52.73%) reported olfactory dysfunction of different levels among 1,627 patients (5). Similar findings were reported by Pang et al. in a meta-analysis of 60 studies including 17,401 COVID-19 patients, where the overall incidence of olfactory dysfunction was 56% (6). In a cohort study at the coronavirus testing center at Technische Universität Dresden, of 34 patients who tested positive for COVID-19, 64.7% of them experienced olfactory loss even before the onset of more typical symptoms of COVID-19, such as fever and cough (7). The above studies suggest that the incidence of anosmia caused by SARS-CoV-2 infection is high and should not be ignored in the diagnosis and treatment of COVID-19 patients.

Besides the reported incidence of olfactory dysfunction in COVID-19 patients, some studies have focused on the regularity of olfactory dysfunction caused by SARS-CoV-2. Yujie et al. conducted a questionnaire survey of 86 COVID-19 patients in Guangzhou Eighth People's Hospital to analyze the development regularity of olfactory dysfunction and found that the average interval from the onset of hyposmia to the onset of symptoms of typical pneumonia was 0.22 ± 4.57 days, while the average duration of hyposmia was 9.09 ± 5.74 days (8). According to the research of Lechien et al., the recovery time of olfactory function in 33% of COVID-19 patients with hyposmia or anosmia (*n* = 357) was 1-4 days, while it was 5-8 days in 39.6% of patients, 9-14 days in 24.2% of patients, and more than 15 days in 3.3% of patients. When limiting their study to patients with anosmia, 20.3% of patients took 1-4 days to recover their olfactory function, 47.5% took 5-8 days to recover their olfactory function, 28.8% took 9-14 days to recover their olfactory function, and 3.4% took more than 15 days to recover their olfactory function (9). In another study of olfactory dysfunction, the authors also found that patients (*N* = 99) lost their olfaction for an average of 8.41 ± 5.05 days (10). Amer et al. also conducted a clinical cohort study of 96 COVID-19 patients with olfactory dysfunction (11), where 80 patients (83%) showed sudden anosmia but only 17% experienced a gradual decrease in olfaction; however, 80% of patients with a gradual decrease in olfaction said they had been in contact with someone with anosmia. These authors also observed that 32 patients (33.3%) had complete olfactory recovery within 1 month, with an average recovery time of 7 days, while 40 patients (41.7%) had partial olfactory recovery and 24 patients (25.0%) achieved no significant recovery. Meini et al. followed up with 100 COVID-19 patients and asked them to rate their recovery of olfactory function on a scale of 100 points, where a score of 80-95 points was considered to suggest a near-complete recovery of olfactory function, and a score of 100 points was considered to indicate a complete recovery of olfactory function. Their final analysis showed that, among patients with subjective symptoms of anosmia (*n* = 29), about 64% had a complete recovery of their olfaction within 1 month and 19% had a near-complete recovery of their olfaction (12). A multicenter prospective study of 138 COVID-19 patients by Vaira et al. reported that the majority of patients showed the most remarkable amelioration in their olfactory dysfunction between 10 and 20 days after COVID-19 onset, with the majority largely recovering within 30 days (13). Considering these studies, it is not difficult to find that olfactory dysfunction is a characteristic clinical manifestation of COVID-19, with an incidence rate of about 56%, and most of the lost olfactory dysfunction can be recovered within 1 month.

In brief, SARS-CoV-2 infection causes olfactory dysfunction, but it may be distinctly different from that demonstrated with URTIs caused by other viruses. Considering the overall incidence rates of olfactory dysfunction, the incidence rate of olfactory dysfunction caused by SARS-CoV-2 is higher than that of olfactory dysfunction caused by a common viral UTRI. The average incidence of olfactory dysfunction caused by SARS-CoV-2 reaches about 55%, or even as high as 80% in some articles and reports, while the average incidence of olfactory dysfunction of other viral UTRIs is about 25%. Seiden et al. reviewed the data of 428 patients with olfactory dysfunction at the Cincinnati University Taste and Smell Clinic, and 78 patients reported a prior UTRI (14). Similar results have also been obtained in other centers. The Connecticut Chemosensory Clinical Research Center found that about 18.6% of 441 post-URTI patients had olfactory dysfunction (15).

Updated studies suggest that infection with SARS-CoV-2 mutant strains, in particular the D164G mutation, may increase the incidence rate of olfactory dysfunction in COVID-19 patients. von Bartheld et al. conducted a systematic review and meta-analysis of studies in South Asian population and found that for the same ethnic group, the incidence of olfactory dysfunction was significantly higher in COVID-19 patients mainly infected with G614 virus strain (31.8%) than in patients mainly infected with D416 virus strain (5.3%) (16). Most of the SARS-CoV-2 mutant strains, such as β (B.1.351), Delta (B.1.617.2), Epsilon (B.1.427 and B.1.429), D164G site mutations were found. In contrast, a cohort study by Brandal et al. from Norway found that only 10 (12%) of 81 patients infected with the SARS-CoV Omicron Variant developed olfactory dysfunction (17) (Table 1).

**Table 1:** Incidence of olfactory dysfunction following infection with COVID-19 and its mutant strains and other upper respiratory viruses.

**Virus types**	**Total no**.	**Gender (male:female)**	**Age (yr)**	**Olfactory dysfuncion (%)**		**Country**	**References**
SARS-CoV-2	155	64:91	Mean (36.3)	35.40%	QS	Turkey	Elibol (4)
SARS-CoV-2	6,635	3617:3007	/	18%	/	China(89%)	Kaur et al. (18)
SARS-CoV-2	86	44:42	Median (25.5)	39.50%	QS	China	Liang et al. (8)
SARS-CoV-2	1,420	458:962	Mean (39.17)	70.20%	QS	Europe	Lechien et al. (10)
SARS-CoV-2	116	58:58	Mean (57.24)	37.90%	QS, VAS	Turkey	Özçelik Korkmaz et al. (19)
SARS-CoV-2	34	/	/	64.70%	QS, VAS	German	Haehner et al. (7)
SARS-CoV-2	103	50:53	Mean (46.8)	61.20%	QS, VAS	Switzerland	Speth et al. (20)
SARS-CoV-2	417	154:263	Mean (36.9)	85.60%	QS	Europe	Lechien et al. (9)
SARS-CoV-2	2,579	1630:949	Mean (44.4)	73.70%	QS, Sniffin'Sticks test	Europe	Lechien et al. (21)
SARS-CoV-2	96	40:56	Mean (34.26)	83%	QS	Egypt	Amer et al. (11)
SARS-CoV-2 with D614G mutation	9,626	5906:3720	Mean (34.32)	31.8%	/	South Asia	von Bartheld et al. (16)
SARS-CoV-2 Omicron variant	81	46:35	Median (36)	12%	QS	Norway	Brandal et al. (17)
URTIV	428	/	/	18%	QS, physical exam	USA	Seiden et al. (14)
URTIV	441	/	Median (53)	18.60%	Physical exam, sniff test	USA	Cain et al. (15)
URTIV	750	336:414	/	26%	QS, sniff test	USA	Deems et al. (22)
URTIV	120	46:74	Median (54.5)	42.50%	physical exam, sniff test	Austria	Quint et al. (23)

*URTIV, upper respiratory tract infection virus; QS, questionnaire survey; VAS, visual analogue scale*.

There are also differences in the onset and duration of olfactory dysfunction caused by SARS-CoV-2 and that caused by other viruses, respectively. Both olfactory dysfunction attributed to COVID-19 and that attributed to other viral URTIs can appear in the incipient stages of viral infection. Olfactory dysfunction in COVID-19 patients can be detected even before the typical symptoms of pneumonia appear, lasts for a shorter time, and the patient recovers more quickly. Although the possibility of olfactory recovery decreases with the duration of olfactory dysfunction, the recovery rates of olfactory dysfunction after COVID-19 tend to be higher (Table 2).

**Table 2:** Characteristics of olfactory dysfunction attributed to COVID-19 vs. other viral UTRIs.

**Characteristic of olfactory dysfunction**	**COVID-19**	**Other URTIs**
Onset	Olfactory dysfunction occurred in 64.7% of COVID-19 patients at an incipient stage or even before other symptoms appeared (7).	At least 33% of patients with post-infection olfactory dysfunction recovered their olfaction, and most of these patients began to recover within the first 6 months after infection (24). A total of 83 patients (31.7%) showed significant improvement in olfactory function at their second doctors' office visiting, approximately 14 months later (25).
	Patients experienced olfactory dysfunction on average 3.4 days after the onset of first symptoms of COVID-19 (21).	
Duration	In COVID-19 patients with hyposmia or anosmia, 96.7% recovered olfaction within 15 days (9).	
	The mean duration of hyposmia was 9.09 ± 5.74 days (8).	
Recovery	There were 32 patients with full olfactory recovery (33.3%), 40 patients with partial olfactory recovery (41.7%), and the remaining 24 patients did not report any significant olfactory recovery (25%) (11).	
	Among patients with subjective symptoms of olfactory dysfunction, about 64% reported full olfactory recovery within 1 month, while 19% reported a nearly full olfactory recovery (12).	

*COVID-19, coronavirus disease 2019; URTI, upper respiratory tract infection*.

## Possible Mechanisms of the Olfactory Dysfunction Caused by SARS-CoV-2

### SARS-CoV-2 Causes Olfactory Dysfunction *via* Nose

There are many different explanations for the mechanism of olfactory dysfunction caused by SARS-CoV-2. Most patients often present with nasal congestion or a runny nose and other acute rhinitis symptoms after experiencing an acute URTI. At that time, the nasal mucosa is swollen because of acute inflammation, capillary dilatation, and an increase in nasal secretions. As a result, if swollen nasal mucosa and secretions block the binding of odorant molecules to olfactory receptors, the patient develops a secondary conductive olfactory dysfunction.

However, a number of studies have shown that rhinitis symptoms caused by inflammation of the nasal mucosa do not seem to be significantly associated with the occurrence of olfactory dysfunction in COVID-19 patients. A prospective questionnaire study by Speth et al. found that 30-50% of 103 COVID-19 patients had nasal congestion and a runny nose, while 61.2% showed olfactory dysfunction, with 14.6% believing that their olfaction decreased and another 46.6% believing that their olfaction was almost completely gone at its worst point. However, the occurrence of olfactory dysfunction was found by univariate analysis to not be significantly associated with concomitant rhinitis symptoms (20). Lechien et al. also found no significant correlation between olfactory dysfunction and rhinitis symptoms after a statistical analysis of 417 patients who rated their own otolaryngological symptoms and COVID-19 severity (9). These studies suggest that infection with SARS-CoV-2 differs from other common viral infections in the area of olfactory dysfunction, and also that COVID-19 may involve olfactory dysfunction through other mechanisms besides mucosal inflammation.

**Figure 1:**
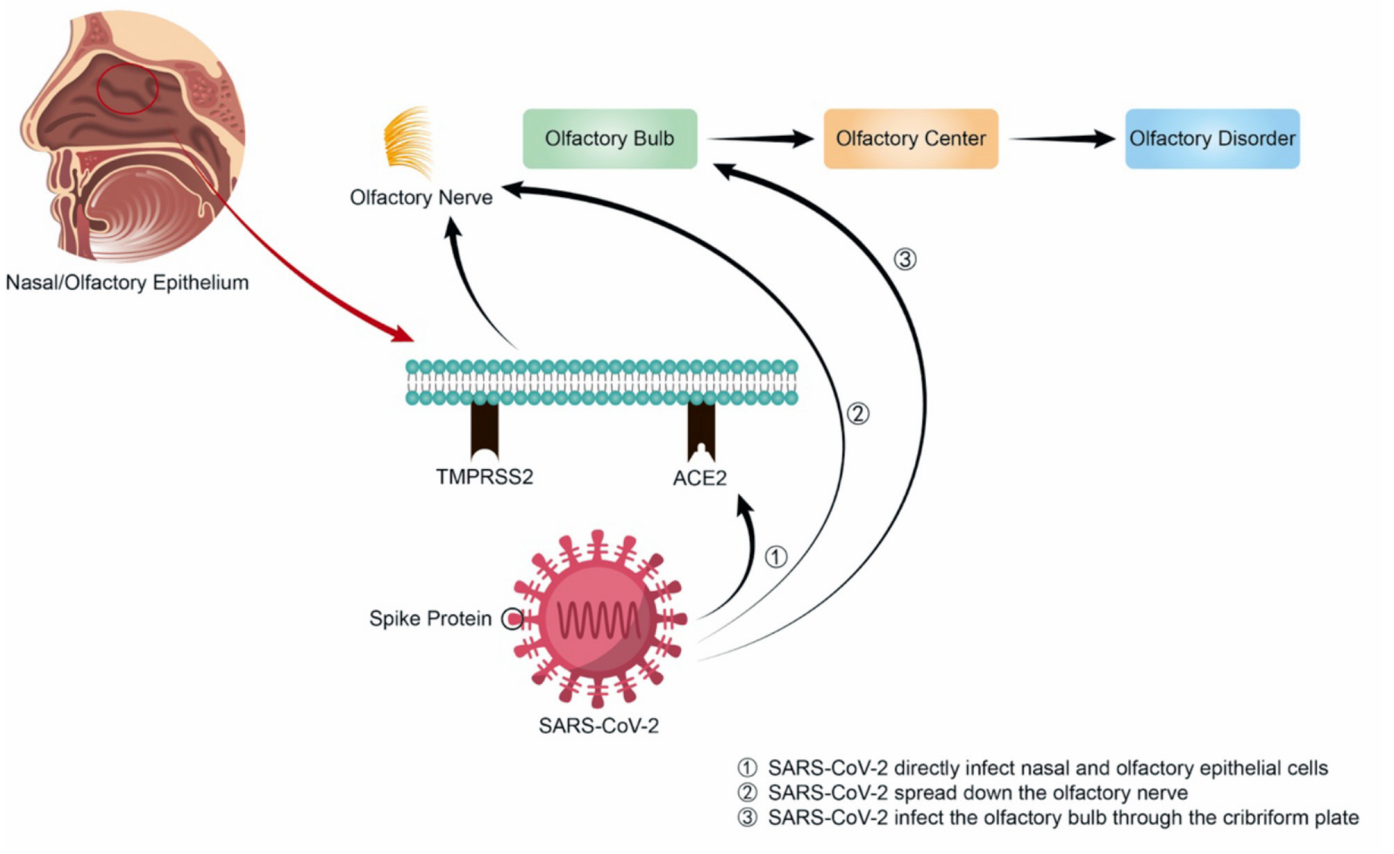
Possible mechanisms of the olfactory dysfunction caused by SARS-CoV-2.

Unlike other upper respiratory viruses, the spike protein of SARS-CoV-2 is first cleaved by TMPRSS2 and interacts with high affinity to ACE2. Then the virus is endocytosed, cleaved by cathepsin L (CSTL), and fused with the endosomal membrane, thereby releasing the single-stranded RNA into the cytoplasm and making further virus replication, assembly, release and further infection and destruction. Therefore, SARS-CoV-2 can directly infect nasal epithelial cells with high expression of ACE2 and TMPRSS2 through the above-mentioned mechanisms (Figure 1). Besides, although the SARS-CoV-2 variants differ in their pathogenicity, they all enter the nasal cavity by binding to the ACE2, causing various symptoms. Some SARS-CoV-2 mutations, such as T478K, may locate in the region interacting with ACE2, enhancing the binding ability of the virus to ACE2 (26), or at the Furin protease cleavage site of spike protein, such as P681R, where the mutation can facilitate the fusion process of SARS-CoV-2 with human cell membrane (27). D614G mutation may enhance the stability of spike protein by inducing conformational changes of spike protein, leading to increased infectivity (16). These mechanisms all contribute to the SARS-CoV-2 infection of cells, increasing the infectivity of the mutant strain, and causing a higher incidence of olfactory dysfunction in COVID-19 patients.

### SARS-CoV-2 Causes Olfactory Dysfunction *via* Central System

In the span of weeks to months, SARS-CoV-2 spread worldwide, similar to the performance of severe acute respiratory syndrome coronavirus (SARS-CoV) in 2003. Both viruses are highly infectious, spread quickly, and primarily colonize the respiratory tract. Similarly, infection with SARS-CoV can also impair olfaction in patients. Gu Jiang, a professor at the Peking University Health Science Center, said at a symposium on SARS-CoV prevention that the virus was found in the nasal mucosa of more than 20 patients during autopsy.

Coronaviruses are neurotropic, as demonstrated in both human and animal models. In 2004, experts detected the RNA of SARS-CoV in the cerebrospinal fluid of a 32-year-old woman in Hong Kong. One year later, SARS-CoV was isolated from the brain tissue of a patient with neurological symptoms (28). The same year, another report also shared that SARS-CoV was detected in the brains of eight patients who died from their infections, and the presence of SARS-CoV RNA was noted in the cytoplasm of many hypothalami and cortical neurons. In 2008, Netland et al. inoculated C57BL/6 transgenic mice intranasally with SARS-CoV and stained for virus antigens to determine the entry site of the virus. These authors ultimately found that the greatest amount of virus antigen was present in the olfactory bulb, which proved that SARS-CoV may enter the body through the olfactory nerve. This is followed by rapid transneuronal spread, eventually making it to the brain (29). Owing to the highly similar structures of SARS-CoV-2 and SARS-CoV, it is speculated that COVID-19 may also involve olfactory dysfunction in this way—namely, SARS-CoV-2 may also be able to spread down the olfactory nerve to the olfactory bulb and the central nervous system, or directly infect the olfactory bulb through the cribriform plate. Then, the virus may follow the olfactory pathway and attack the olfactory cortex in the frontal lobe of the brain, which is answerable for olfaction. However, olfactory dysfunction resulting from SARS-CoV-2 infection is not caused by direct damage to the central nervous system; if it were, the onset and recovery of olfactory dysfunction would be slower and the symptoms would likely be more complex. Thus, the mechanism of olfactory dysfunction in COVID-19 may differ from that of other viruses; not only is secondary conducive olfactory dysfunction caused by acute inflammation after infection with other viruses, but other mechanisms also participate. Previous studies have found that olfactory sensory neurons in the olfactory epithelium of mice infected with H1N1 were reduced in number, the olfactory epithelium became thinner, and the expression level of olfactory marker protein messenger RNA decreased, suggesting that this may be one of the pathogeneses of olfactory dysfunction after virus infection (30). Furthermore, the literature has reported that other viruses may reduce the number of olfactory tracts and cause the loss of olfactory receptor cilia after URTI. Some of the olfactory epithelium may be replaced by respiratory epithelium, or there may be extensive scarring (31, 32)—that is, part of the olfactory epithelium might lose its original characteristics during infection, which may lead to olfactory dysfunction.

It is reported that SARS-CoV-2 can infect immune-privileged sites like eyes and brain. For example, a female COVID-19 patient with glaucoma underwent two NEGATIVE RT-PCR tests after conventional treatment, but SARS-CoV-2 RNA was detected again in her upper respiratory tract and aqueous humor 2 months later (33). Solomon et al. found SARS-CoV-2 specific antigens and RNA in 3 medulla sections, 3 lobe, and 3 olfactive nerve sections on the autopsies of 18 dead COVID-19 patients (34). The central system is also one of the immune-privileged parts of the human body, so we speculate that SARS-CoV-2 may also persist in the central system, which makes that a small number of patients may not recover significantly from olfactory dysfunction. The way how the virus gets into the brain is still to be explored.

Other scholars have speculated, from the perspective of molecular biology, that SARS-CoV-2 may enter the central nervous system by infecting peripheral tissues through the cellular mechanism of active transport, leading to secondary olfactory damage (35). According to the research of Ibekwe et al., SARS-CoV-2 may also directly attack and damage olfactory receptors, thus inhibiting the transmission of odor signals. This can lead to temporary or permanent anosmia (36). In addition, some scholars believe that drug treatment and the use of a large number of disinfectants may lead to olfactory dysfunction during COVID-19.

## Treatments for Olfactory Dysfunction Attributed to Viral Infection

At present, there are few reports on the treatment of olfactory dysfunction caused by SARS-CoV-2. In view of the similarities between the mechanism of olfactory dysfunction caused by SARS-CoV-2 and that of olfactory dysfunction caused by other viruses, this study briefly covers literature on the treatment of olfactory dysfunction caused by other viruses for reference.

### Drug Therapy

For viral URTIs, including COVID-19, if the olfactory dysfunction is caused by acute inflammation, such as nasal mucosal swelling, glucocorticoids can be applied inside the nasal cavity or systemically to treat the olfactory dysfunction. Glucocorticoids are known to regulate the body's immune response in cells and transcription; at the cellular level, they can cause apoptosis of T lymphocytes, neutrophils, basophils, and eosinophils to reduce inflammation, while, at the transcriptional level, glucocorticoids can repress a plethora of cytokines, chemokines, cell-adhesion molecules, inflammatory enzymes, and the pro-inflammatory genes of receptors to resolve the inflammatory process (37). The systemic use of glucocorticoids may be more effective than topical administration. Seiden et al. reported that topical hormone therapy improved olfactory dysfunction in 25% of patients, while systemic hormone therapy was effective in 83% of patients with olfactory dysfunction (14). However, the effect of glucocorticoids on nervous olfactory dysfunction caused by the impairment of the olfactory epithelium and olfactory nerve is not ideal.

The cells of the olfactory nerve are capable of regeneration; thus, drug therapy that can support and promote olfactory nerve regeneration may be beneficial for olfactory recovery. Retinoic acid (RA), a metabolite of vitamin A, is a significant transcriptional regulator of tissue development and regeneration. Studies have found that RA signaling is activated during embryonic development of the olfactory system and neuron regeneration in adults (38), suggesting that the application of vitamin A inside the nose may be beneficial in the treatment of post-infection neural olfactory dysfunction. Meanwhile, lipoic acid, a B vitamin, is a powerful antioxidant and is thought to be suitable for treating nerve damage involving free radicals. Hummel et al. provided alpha-lipoic acid daily to 23 patients with post-URTI olfactory dysfunction and found that 14 patients (61%) experienced improved olfactory dysfunction after treatment, including 8 who reported significantly improved olfactory function (39). In addition, adequate vitamin C and E intake may also help restore olfactory nerves' function by protecting nerve cells or promoting recovery. Other literature has reported that treatment with sodium citrate (40), zinc (41), and caroverine (42) may have a certain effect on the recovery of patient olfactory function.

### Olfactory Training Therapy

Olfactory training is a therapy during which patients actively and repeatedly sniff various types of odors to improve their olfactory function. In 2009, Hummel et al. found that olfactory function improved in about 30% of patients over a 12-week period of olfactory training compared to those who did not participate in olfactory training, and olfactory training was effective not only in patients with post-URTI olfactory dysfunction but also in patients with post-traumatic traumatic or idiopathic olfactory dysfunction (43). Damm et al. also believed that repeated short-term exposure to different odors may modulate the regenerative capacity of the olfactory mucosa, leading to the regeneration and increased expression of olfactory receptor neurons, thus enhancing olfactory sensitivity (44). In addition, increasing the types of odors and prolonging the period of olfactory training may also improve the success rate of olfactory dysfunction treatment (45).

## Summary and Prospects

Although SARS-CoV-2 belongs to the same coronavirus family as SARS-CoV, both the transmissibility and infectivity of the former are stronger. Many studies have shown that SARS-CoV-2 infection can lead to olfactory dysfunction, and many scholars believe that neurosensory symptoms, such as olfactory dysfunction, can be used as a diagnostic maker for the early diagnosis of COVID-19. The symptoms of olfactory dysfunction may also be considered as part of routine screening for COVID-19 (8).

In this study, the characteristics of olfactory dysfunction caused by SARS-CoV-2 infection are analyzed and summarized. The incidence of olfactory dysfunction caused by SARS-CoV-2 infection is relatively higher, but the average duration of olfactory dysfunction or anosmia is shorter and the possibility of olfactory recovery is higher compared to the post-URTI olfactory dysfunction caused by other common viruses. These differences may be related to the unique structure, high infectivity and mutation sites of the SARS-CoV-2 and the variations in the main mechanism of olfactory dysfunction caused by SARS-CoV-2 and by the other upper respiratory viruses.

It is worth noting that the SARS-CoV-2 is mutating. The Omicron variant is currently sweeping the world, but studies have found that COVID-19 patients infected with Omicron variant are significantly less likely to develop olfactory dysfunction, exhibiting symptoms indistinguishable from those of influenza. Its reason may be that extensive vaccination has caused patients infected with the Omicron variant to develop only mild symptoms, or even asymptomatic infection. Therefore, the public should strengthen prevention and control of the pandemic and vaccination.

The treatment of olfactory dysfunction in COVID-19 patients has been poorly reported to date. At present, there is no clear consensus or guidelines for the treatment of olfactory dysfunction after viral infection caused by other mechanisms, except for conductive olfactory dysfunction caused by nasal mucosal swelling or nasal obstruction caused by acute inflammation. Vitamins, zinc, calcium channel blockers, and olfactory rehabilitation training may be effective as therapeutic options in the treatment of other olfactory dysfunction caused by other viral infection, and their effects on the olfactory dysfunction caused by SARS-CoV-2 infection still need to be confirmed by randomized controlled trials.

In conclusion, SARS-CoV-2 infection can lead to olfactory dysfunction in patients, albeit with an earlier onset, shorter duration, and higher incidence and recovery rates compared to other viral infections. Olfactory functions are very important to human life, so medical personnel should adopt careful measures of investigating, understanding, and identifying olfactory dysfunction, and actively offer corresponding treatment measures.

## Author Contributions

MX substantial contributions to the design of the work and approval of the final version of this article. YM, ZS, BY, CF, JW, BW, and YS participated in the conception and design of the study, collected the literature, prepared the tables, and wrote the manuscript. All authors agree to be accountable for all aspects of the work in ensuring that questions related to the accuracy or integrity of any part of the work are appropriately investigated and resolved.

## Funding

This study was supported by grants from the Cultivation Project of the Major Research Plan of the National Natural Science Foundation of China (Grant No. 91949119), the National Natural Science Foundation of China (Grant Nos. 81800902, 82101209, and 82101212), the Science and Technology Commission of Shanghai Municipality (Grant No. 21ZR1440200), the Shanghai Sailing Program (Grant Nos. 20YF1426400 and 19YF1430300), and Ruijin Youth NSFC Cultivation Fund.

## Conflict of Interest

The authors declare that the research was conducted in the absence of any commercial or financial relationships that could be construed as a potential conflict of interest.

## Publisher's Note

All claims expressed in this article are solely those of the authors and do not necessarily represent those of their affiliated organizations, or those of the publisher, the editors and the reviewers. Any product that may be evaluated in this article, or claim that may be made by its manufacturer, is not guaranteed or endorsed by the publisher.
